# Simultaneous Estimation of Amlodipine Besilate and Olmesartan Medoxomil in Pharmaceutical Dosage Form

**DOI:** 10.4103/0250-474X.58190

**Published:** 2009

**Authors:** S. B. Wankhede, S. B. Wadkar, K. C. Raka, S. S. Chitlange

**Affiliations:** Department of Pharmaceutical Chemistry, Pad. Dr. D. Y. Patil Institute of Pharmaceutical Sciences and Research, Sant Tukaram Nagar, Pimpri, Pune-411 018, India

**Keywords:** Amlodipine besilate, area under curve method, olmesartan medoxomil, reverse phase high performance liquid chromatography, simultaneous equation method

## Abstract

Two UV Spectrophotometric and one reverse phase high performance liquid chromatography methods have been developed for the simultaneous estimation of amlodipine besilate and olmesartan medoxomil in tablet dosage form. First UV spectrophotometric method was a determination using the simultaneous equation method at 237.5 nm and 255.5 nm over the concentration range 10-50 μg/ml and 10-50 μg/ml, for amlodipine besilate and olmesartan medoxomil with accuracy 100.09%, and 100.22% respectively. Second UV spectrophotometric method was a determination using the area under curve method at 242.5-232.5 nm and 260.5-250.5 nm over the concentration range of 10-50 μg/ml and 10-50 μg/ml, for amlodipine besilate and olmesartan medoxomil with accuracy 100.10%, and 100.48%, respectively. In reverse phase high performance liquid chromatography analysis carried out using 0.05M potassuim dihydrogen phosphate buffer:acetonitrile (50:50 v/v) as the mobile phase and Kromasil C18 (4.6 mm i.d.×250 mm) column as the stationery phase with detection wavelength of 238 nm. Flow rate was 1.0 ml/min. Retention time for amlodipine besilate and olmesartan medoxomil were 3.69 and 5.36 min, respectively. Linearity was obtained in the concentration range of 4-20 μg/ml and 10-50 μg/ml for amlodipine besilate and olmesartan medoxomil, respectively. Proposed methods can be used for the estimation of amlodipine besilate and olmesartan medoxomil in tablet dosage form provided all the validation parameters are met.

Amlodipine besilate (AMLO), chemically, [3-ethyl-5-methyl(4RS)-2-[(2-aminoethoxy) methyl]-4-(2-chlorophenyl)-methyl-1-dihydropyridine-3,5-dicarboxylate benzenesulfonate[1], is a long acting calcium channel blocker used which is used as an antihypertensive agent[2–4]. Olmesartan medoxomil (OLME), chemically, 2,3-dihydroxy-2-butenyl 4-(1-hydroxy-1-methylethyl)-2-propyl-1-[*p*-(*o*-1*H*-tetrazol-5-ylphenyl)benzyl]imidazole-5-carboxylate, cyclic-2,3-carbonate, is an angiotensin II receptor blockers used as an antihypertensive agent[5]. AMLO is official in BP[1], whereas OLME is not official in any pharmacopoeia. Both the drugs are marketed as combined dose tablet formulation in the ratio of AMLO:OLME 05:20 mg. Literature survey revealed that a number of methods have been reported for estimation of AMLO[6–15] and OLME[16–17] individually or in combination with other drugs. However, there is no analytical method reported for the simultaneous estimation of AMLO and OLME in a combined dosage formulation. Present work describes three simple, accurate, reproducible, rapid and economical methods for simultaneous estimation of AMLO and OLME in tablet formulation. A double-beam Shimadzu UV/Vis spectrophotometer, 1700 Pharmaspec, with spectral bandwidth of 2 nm, wavelength accuracy of ±0.5 nm and a pair of 1-cm matched quartz cells, was used to measure absorbance of the resulting solution. A gradient reverse phase high performance liquid chromatography (Merck Hitachi) with L-7100 double reciprocating pump, L-7400 UV detector, and Kromasil C18 (4.6 mm i.d.×250 mm) column as the stationery phase was used. The RP-HPLC system was equipped with Winchrom software for data processing. Standard gift sample of AMLO was provided by Glennmark Pharmaceuticals Ltd, Nashik, India and OLME by Macleods Pharmaceuticals Ltd, Mumbai, India. AMLO and OLME combination tablets (Olmezest-AM, 05 mg amlodipine besilate and 20 mg olmesrtan medoxomil; manufacture by SUN Pharmaceutical Industries, Dadra, India), were purchased from the local pharmacy. For UV-Spectrophotometry, methanol of analytical grade was used as solvent, standard stock solutions of AMLO (100 μg/ml) and OLME (100 μg/ml) were prepared in methanol and used for the analysis. For RP-HPLC, acetonitrile of HPLC grade was used, a buffer solution of 50 mM was prepared. A mixture of phosphate buffer and acetonitrile in the ratio of 50:50 v/v was used as mobile phase and was filtered before use through 0.45 μ membrane filter. Kromasil C18 column (4.6 mm i.d.×250 mm) was used as stationary phase. Constant flow of 1.0 ml/min was maintained throughout the analysis. Detection was carried out using UV detector at 238 nm. Standard stock solutions of AMLO (100 μg/ml) and OLME (100 μg/ml) were prepared in mobile phase and used for the analysis.

For the selection of analytical wavelength for the simultaneous equation method (method-A), solutions of AMLO and OLME (20 μg/ml, each), were prepared separately by appropriate dilution of standard stock solution and scanned in the spectrum mode from 200 nm to 400 nm. From the overlain spectra of both drugs (fig. 1), wavelengths 237.5 nm (λ_max_ of AMLO) and 255.5 nm (λ_max_ of OLME) were selected for the simultaneous equations. Calibration curves for AMLO and OLME were prepared in the concentration range of 10-50 μg/ml and 10-50 μg/ml at both the wavelengths, respectively. The absorptivity values were determined for both the drugs at both the wavelengths and following Eqns were used, A_1_=41.86C_AMLO_+38.99C_OLME_ (1) and A_2_=28.25C_AMLO_+41.39C_OLME_ (2), where A1 and A2 are absorbances of the sample at 237.5 nm and 255.5 nm, respectively, 41.86 and 28.25 are absorptivities of AMLO at 237.5 nm and 255.5 nm, respectively, 38.99 and 41.39 are the absorptivities of OLME at 237.5 nm and 255.5 nm, respectively. C_AMLO_ is the concentration of AMLO and C_OLME_ is the concentration of the OLME. The mixture concentration was determined by using the Eqns 1 and 2.

**Fig. 1:**
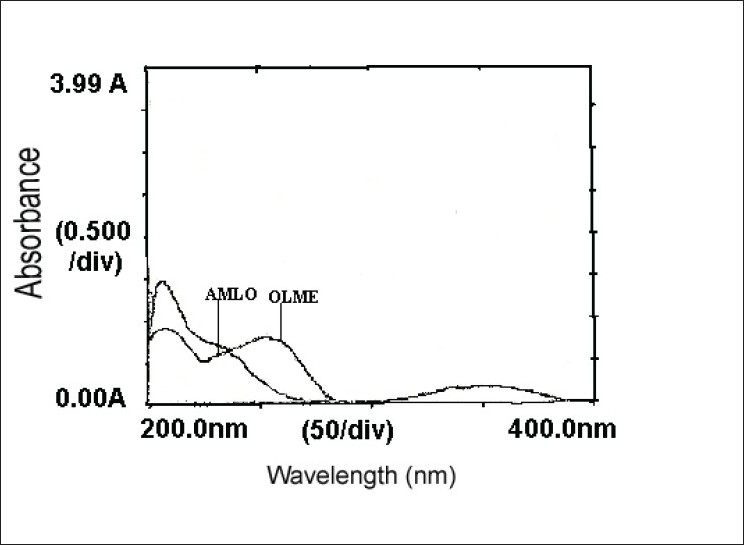
Overlain spectra of AMLO and OLME AMLO is amlodipine besylate and OLME is olmesartan medoxomil

In the area under curve method (method-B), from the overlain spectra of both drugs (fig. 1), wavelengths range 242.5-232.5 nm (for AMLO) and 260.5-250.5 nm (for OLME) were selected for the analysis. The calibration curves for AMLO and OLME were prepared in the concentration range of 10-50 μg/ml and 10-50 μg/ml at both the wavelength range, respectively. The absorptivity values were determined for both the drugs at both the wavelength range and following Eqns were used, A_1_ = 414.13C_AMLO_+389.32C_OLME_ (3) and A_2_ = 283.08C_AMLO_+408.97C_OLME_ (4), where A1 and A2 are area under curve of the sample at 242.5-232.5 nm and 260.5-250.5 nm, respectively, 414.13 and 283.08 are absorptivities of AMLO at 242.5-232.5 nm and 260.5-250.5 nm, respectively, 389.32 and 408.97 are the absorptivities of OLME at 242.5-232.5 nm and 260.5-250.5 nm, respectively. C_AMLO_ is the concentration of AMLO and C_OLME_ is the concentration of the OLME. The mixture concentration was determined by using the Eqns 3 and 4.

In the reverse phase high performance liquid chromatography (method-C), standard stock solution of AMLO and OLME (1000 μg/ml) was prepared in mobile phase separately. The wavelength selected for analysis is 238 nm. The calibration curves for AMLO and OLME were prepared in the concentration range of 04-20 μg/ml and 10-50 μg/ml, respectively at 238 nm. Calibration curve was constructed by plotting concentration against peak area.

In the UV spectrophotometric method, for estimating AMLO and OLME in commercial formulations, twenty tablets were weighed and average weight was calculated. The tablets were crushed to obtain a fine powder. Tablet powder equivalent to 5 mg of AMLO was transferred to 25.0 ml volumetric flask containing 20.0 ml methanol and exposed to ultrasonic radiations for 20 min and then final volume was made up to the mark with methanol. The solution was then filtered through a Whatmann filter paper No. 41. The filtrate was appropriately diluted with the same solvent to obtain final concentrations of 8 μg/ml for AMLO and 32 μg/ml for OLME. Concentrations of both AMLO and OLME were determined by measuring the absorbance of the sample at 237.5 and 255.5 nm (method-A) and at 242.5-232.5 nm and 260.5-250.5 nm (method B) in the spectrum mode and values were substituted in the respective formulae to obtain concentrations. Results of the tablet analysis were analysed against the calibration curve in quantitation mode.

In the RP-HPLC method, for estimating AMLO and OLME in commercial formulation, tablet sample solution containing 8 μg/ml of AMLO and 32 μg/ml of OLME was prepared in similar manner as described under UV spectrophotometric method using mobile phase as diluent instead of methanol. The diluted solutions were filtered through 0.20 μ filter. Twenty microlitres of solutions were injected and chromatographed under above mentioned chromatographic conditions. A typical chromatogram of AMLO and OLME is shown in (fig. 2). The concentration of both AMLO and OLME was determined by comparing peak area of sample with that of standard at 238 nm. The results of tablet analysis are shown in Table 1.

**Fig. 2:**
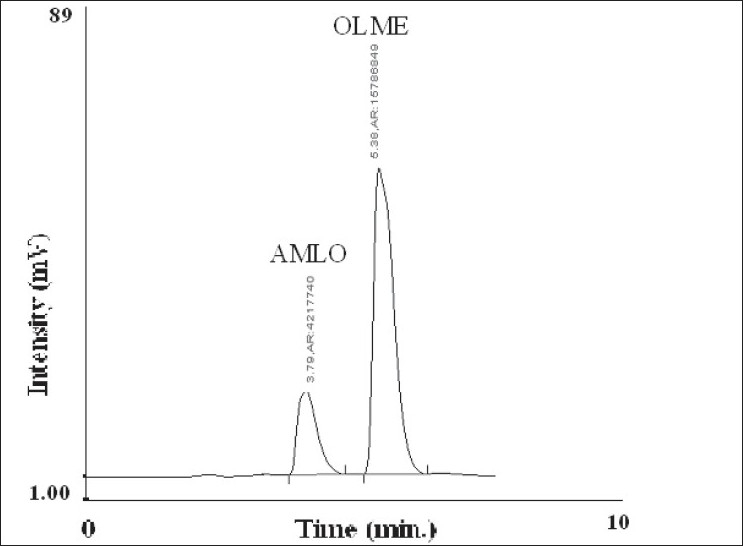
Typical chromatogram of AMLO and OLME AMLO is amlodipine besylate and OLME is olmesartan medoxomil

**TABLE 1:** ANALYSIS OF TABLET FORMULATION

Method	Component	Label Claim (mg/tab)	Amount Found	Estimated Label Claim*	SD	CV
A	AMLO	05	5.012	100.27	0.1752	0.1747
	OLME	20	20.091	100.46	0.2921	0.2920
B	AMLO	05	5.01	100.10	0.1732	0.1730
	OLME	20	20.07	100.36	0.2134	0.2126
C	AMLO	05	5.012	100.25	0.6136	0.6120
	OLME	20	20.065	100.32	0.2785	0.2776

* Average of six determinations, SD denotes standard deviation and CV denotes coefficient of variation.

The proposed chromatographic system was found suitable for effective separation and quantitation of AMLO (RT-3.79 min) and OLME (5.39 min). The system suitability parameters were found to be, resolution-2.22, tailing factor- 1.30 for AMLO and 1.17 for OLME. Recovery studies were carried out by standard addition method at three different levels 80, 100 and 120%. The % recovery of AMLO and OLME in the sample mixture was determined. The results of recovery studies obtained by proposed method were validated by statistical evaluation and are recorded in Table 2.

**TABLE 2:** RESULTS OF RECOVERY STUDIES

Level of Recovery (%)	Amt. of Pure Drug Added (mg)		Method-A % Recovery		Method-B % Recovery		Method-C % Recovery	
			
	AMLO	OLME	AMLO	OLME	AMLO	OLME	AMLO	OLME
80	04	16	100.71	100.08	100.07	100.57	100.01	100.10
100	05	20	100.54	100.42	100.18	100.32	100.08	100.17
120	06	24	99.04	100.15	100.07	100.54	99.85	99.92
Mean % Recovery			100.10	100.22	100.11	100.48	99.98	100.06
SD			0.8422	0.1743	0.2575	0.1414	0.1685	0.1202
CV			0.8414	0.1739	0.2572	0.1407	0.1682	0.1201
SE			0.3439	0.0712	0.1051	0.0577	0.0688	0.0491

SD is the standard deviation, CV is the coefficient of variation and SE is the standard error.

The methods discussed in the present work provide a convenient and accurate way for simultaneous analysis of AMLO and OLME. Percent label claim for AMLO and OLME in tablet, by all the methods, was found in the range of 98.57 to 101.56 %. Standard deviation and coefficient of variance for six determinations of tablet sample, by these methods, was found to be less than ±2.0 indicating the precision of the methods. Accuracy of proposed methods was ascertained by recovery studies and the results are expressed as % recovery. Percent recovery for AMLO and OLME, by all three methods, was found in the range of 99.01 to 100.97%, values of standard deviation and coefficient of variation were in the range of ±0.1202 to ±0.8422 and 0.1201 to 0.8414, respectively indicating the accuracy of both the methods. Based on the results obtained, it is found that the proposed methods are accurate, precise, reproducible and economical and can be employed for routine quality control of AMLO and OLME in combined dose tablet formulation.
